# All-in-One Electric Double Layer Supercapacitors Based on CH_3_NH_3_PbI_3_ Perovskite Electrodes

**DOI:** 10.1021/acsomega.2c06664

**Published:** 2022-12-09

**Authors:** Seher Güz, Merve Buldu-Akturk, Hasan Göçmez, Emre Erdem

**Affiliations:** † Faculty of Engineering, Department of Metallurgy and Materials Engineering, Dumlupınar University, Kütahya43100, Turkey; ‡ Faculty of Engineering and Natural Science, Sabanci University, İstanbul34956, Turkey; § Integrated Manufacturing Technologies Research and Application Center & Composite Technologies Center of Excellence, Sabanci University, Teknopark Istanbul, Pendik, 34906Istanbul, Turkey; ∥ Center of Excellence for Functional Surfaces and Interfaces for Nano-Diagnostics (EFSUN), Sabanci University, Orhanli, Tuzla, 34956Istanbul, Turkey

## Abstract

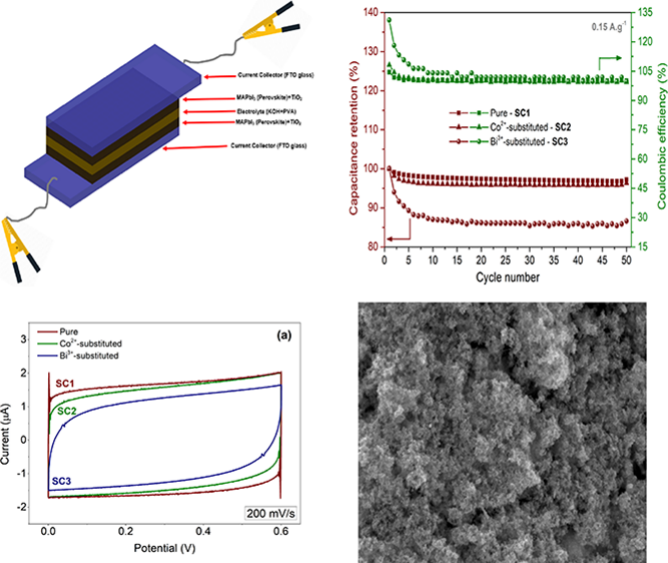

Supercapacitors (SCs) are widely used energy storage devices in various applications that require instantaneous power supply and fast response times; however, the challenge for achieving high performance demands the continuous development and tailoring of electrode materials. Organic–inorganic halide perovskites (OIHPs) have recently received significant attention in electrochemical energy storage and conversion applications due to their unique properties including high charge carrier mobility, high mixed (electronic-ionic) conductivity, and presence of large oxygen vacancies. This study presents the fabrication and use of OIHPs based on methyl-ammonium lead iodide (CH_3_NH_3_PbI_3_) and its Co^2+^- and Bi^3+^-substituted derivatives (CH_3_NH_3_Pb_1–*x*_Co_*x*_I_3_ and CH_3_NH_3_Pb_1–*x*_Bi_*x*_I_3_, respectively, where *x* = 0.1) as electrodes for SCs. SC devices were constructed symmetrically by sandwiching the synthesized electrode materials in a quasi-solid-state electrolyte between two TiO_2_-coated FTO glasses. We discussed the optimization parameters (i.e., A-site doping, B-site doping, and controlling the stoichiometry of the anion and cation) to improve the electrochemical performance of the fabricated SCs. Furthermore, the effects of substitution ions (Co^2+^ and Bi^3+^) on the charge–discharge performance, energy and power density, defects, crystallinity, and microstructure were demonstrated. Electrochemical performances of the electrodes were analyzed by using CV, EIS, and GCPL techniques. The highest power density of 934.6 W/kg was obtained for Bi-substituted perovskite electrodes. Fabricated SC devices show good cyclability with 97.2, 96.3, and 86.6% retention of the initial capacitances after 50 cycles for pure, Co^2+^-substituted, and Bi^3+^-substituted perovskite electrodes, respectively.

## 1 Introduction

The rapid increase in population and industrialization are causing increasing pressure on the available energy sources; hence, studies for the development of renewable energy production and storage technologies are rapidly increasing worldwide. Supercapacitors (SCs) are energy storage devices that exhibit various important properties including rapid power supply, fast charge–discharge rates, and long cycle life. SCs can be classified into three main categories based on their charge storage mechanism: (1) electric double layer capacitors (EDLCs), (2) pseudocapacitors, and (3) hybrid SCs.^1^ In EDLC, electrical energy is stored electrostatically by the accumulation of charges at the electrode and electrolyte interface. During the charging process, electrons flow from the negative electrode to the positive one through the external circuit, while the cations and anions flow toward the negative and positive electrodes, respectively. In pseudocapacitors, charge storage involves the Faradaic reactions.^2^ Hybrid SCs are formed when both mechanisms are combined. Due to their mixed (ionic–electronic) conductivity and high charge carrier mobility properties, perovskite materials are promising materials for use as electrodes for SCs.

An ideal electrode for SC should exhibit high specific capacity and specific capacitance. The materials to be preferred for efficient SCs should have good electronic and ionic conductivity, high surface area, and porous structure. Due to its tailorable porous structure and good electrochemical and physical properties, thin film TiO_2_ is one of the most suitable charge transport materials. Many metal oxides, such as TiO_2_ used in electrochemical energy storage systems, have low surface area, and therefore, recent research has focused on metal oxide integrated metal–organic frameworks (MOFs).^3^ Metal–organic frameworks (MOFs) are new type porous organic–inorganic hybrid materials.^4^ Also, MXenes can be used in supercapacitors with high specific surface areas and have the ability to trap electrons.^5^ MXenes are a kind of 2D transition metal carbides, carbonitrides, and nitrides.^6^

Not only the TiO_2_ layer but also the interface between TiO_2_ and perovskite has an impact on the device performance, which is strongly affected by the surface oxygen vacancies. Notably, oxygen-deficient TiO_2_ samples possess excellent conductivity performance by oxygen vacancies.^7^ The most common point defects in TiO_2_ are the oxygen vacancies (Ti^3+^-V_O_), which can transform into trap states (Ti^4+^-V_O_) upon excitation.^8−10^

The major ternary structural families of perovskites are A_2_BO_4_, AB_2_X_4_, and ABX_3_. A_2_BO_4_ is a layered perovskite structure and exhibits a Ruddlesden–Popper phase type that has a two-dimensional perovskite structure.^11^ Its general formula is A_*n* + 1_B_*n*_X_3*n* + 1_, where A and B sites refer to cation species and X refers to anion species. AB_2_X_4_ is a spinel structure, where A, B, and X are a magnetic cation, nonmagnetic cation, and chalcogen ions such as O^2–^, Se^2^, or S^2–^, respectively. Both the A_2_BO_4_ oxides and spinel structures are suitable catalyst materials due to their high catalytic activities.^12,13^ ABX_3_ belongs to the family of CaTiO_3_ minerals. In this formula, region A means organic or inorganic large cations, region B stands for divalent metallic cations (i.e., Mn^2+^, Cu^2+^, Co^2+^, Mg^2+^, Ni^2+^, Sn^2+^, Pb^2+^, Bi^3+^, and Eu^2+^), and the X site contains halide ions (Cl^–^, Br^–^, I^–^).^14^ Previous reports show that the Co^2+^ substitution provides new dimensions for tuning the electronic and crystallographic properties of perovskite materials while maintaining the photovoltaic performance.^15^ Therefore, Co^2+^ ions can be substituted in the B region of the perovskite structure and mediate the alteration of the crystal phase. Moreover, the stability of the perovskite structure is increased by substituting the Pb^2+^ cation with Bi^3+^, which is a non-toxic 6p-block element.^16^ Due to these improvements in the perovskite structure, Co^2+^ and Bi^3+^ ions were selected as substitution ions for Pb^+2^ in this study. The inorganic constituents of perovskites can be tailored due to their ability to adapt to various sizes and to have different properties. The fabrication process is facile, and the stability of a perovskite structure can be estimated by using the well-known Goldschmidt tolerance factor, which is expressed by the formula *t* = (*R*_A_ + *R*_X_) /√2(*R*_B_ + *R*_X_). Here, *R*_A_, *R*_B_, and *R*_X_ are the ionic radii of A, B, and X, respectively. An ideal perovskite structure has a cubic system with *t* = 1.^17^ In our study, it was determined that the Bi^3+^ (rBi^3+^ = 117 pm) and the Co^2+^ (rCo^2+^ = 70 pm) ions could be substituted for the Pb^2+^ion based on the Goldschmidt tolerance factor (*t*). The “*t*” tolerance factors were calculated as 0.91 and 1.06 for these elements, respectively. According to the Goldschmidt’s empirical rules for element substitution, perfect substitution can be achieved if the ionic radius is less than 15%, but limited substitution can occur if the size differs between 15 and 30%. The ionic radii difference between Pb^+2^ and Bi^+3^ is 11.36%, indicating a perfect substitution.^18^ In the density functional theory (DFT) calculations, it was determined that the organic halogen perovskite compounds CH_3_NH_3_Pb_(1–*x*_)Bi_*x*_I_3_, which is formed by the substitution of nonstoichiometric Pb^2+^ ions with Bi^3+^ ions, will be more efficient in terms of environmental safety and solar energy conversion capacity.^19^ On the other hand, Co^2+^ has a smaller ionic radius than Pb^2+^, which effectively lightens the Pb–I–Pb tilting bond, leading to an increase in electrical conductivity without changing the thin film morphology, thus making it a suitable substitution ion for perovskite-based electrodes for SCs.^20^ After the partial substitution of Pb^2+^ by Co^2+^, the CH_3_NH_3_Pb_0.9_Co_0.1_I_3_ film exhibits a larger crystal size and enlarged gaps between the large crystal field, hence causing a significant leakage current.^21^

Electrolytes play an important role in SCs as they provide charge balance and charge transfer between the two electrodes.^22^ Generally, aqueous electrolytes (KOH, NaOH, LiOH, Na_2_SO_4_, H_2_SO_4_, (NH_4_)_2_SO_4_, K_2_SO_4_, Li_2_SO_4_, MgSO_4_, CaSO_4_, BaSO_4_, KCl, NaCl, LiCl, HCl, CsCl, CaCl_2_, KNO_3_, LiNO_3_, Na(CH_3_COO), Li(CH_3_COO), Mg(CH_3_COO)_2_, Na_2_HPO_4_, NaHCO_3_, Na_2_B_4_O_7_) are used in SCs for high capacitance and improved conductivity, but they give lower power density compared to the solid ones.^23^ The solid-state electrolytes such as polyvinylidene fluoride (PVDF), polyethylene oxide (PEO), polymethyl methacrylate (PMMA), Li salts, polyvinyl chloride (PVC), and silicon dioxide (SiO_2_) can prevent the leakage problems, but they have low conductivity and high viscosity.^24−31^ Therefore, quasi-solid-state gel electrolytes would be the best option for the present study. Polyvinyl alcohol (PVA) gel is generally used with ionically conducting agents such as H_3_PO_4_, KOH, and other salts to form quasi-solid-state electrolytes for the EDLCs.^32−34^ The key factor to use the PVA gel electrolyte here is to prevent electrolyte leakage that can commonly occur in EDLCs.^35^

Research has focused on other materials such as layered nanoclays, which can be used in all components such as electrolytes, electrodes, and separators in the combination of supercapacitors, with quite a lot of reserves. However, layered structures such as nanoclays are difficult to understand and not every nanoclay structure is used in energy storage systems. So, advanced measurement and theoretical calculation will make it easier to understand.^36^

In this work, perovskite-based active electrodes are fabricated by the two-step solution process. Environment-friendly and high stability Bi^3+^ ions were chosen for the substitution of Pb^2+^ ions in perovskite-based SCs. The effects of Bi^3+^ substitution on the film formation and electrochemical property of perovskites were studied. In addition, Co^2+^ ions were chosen to substitute the Pb^2+^ ions to observe the electronic and crystallographic tunability. Furthermore, a quasi-solid-state electrolyte was synthesized and used in the SCs for improving the device performance. Finally, an effective design of all-in-one SC devices was introduced, and their electrochemical performances were tested by using CV, EIS, and GCPL techniques.

## 2 Materials and Methods

### 2.1 Materials

Lead(II) iodide (PbI_2_ purity of 99.99%), bismuth(III) iodide (BiI_3_ 99%), and cobalt(II) iodide (CoI_2,_ 95%) were used as Pb^2+^, Co^2+^, and Bi^3+^ sources were purchased from Sigma Aldrich. 2-Propanol was used as a solvent (99.8%) and was purchased from Sigma Aldrich. Methylammonium iodide (MAI, 99%) was purchased from Lumtec and used as an organic part in perovskite. Polyvinyl alcohol (PVA) was obtained from Merck and was used as a gelling agent. Fluorine-doped tin oxide (FTO, TCO30-8) glass was obtained from Solaronix and was used as front contact. Anhydrous *N*,*N*-dimethylformamide (DMF) was obtained from J.T Baker and was used as a PbI_2_ solvent. Acetone (99.5%) was used as a stabilizer, titanium(IV) isopropoxide (98 + %) was used to obtain TiO_2_, and ethylene glycol (99.8%) was used as a solvent and purchased from Gainland Chemical Company, Acros Organics, and VWR Chemicals, respectively. Citric acid monohydrate (≥99.5%) was used as a chelate agent, potassium hydroxide (KOH) was used as a conducting agent, and they were obtained from Tekkim and DETSAN, respectively.

### 2.2 Synthesis

#### 2.2.1 Preparation of TiO_2_ Thin Film

First, FTO-coated glass substrates were cleaned by ultrasonication in deionized water, ethanol, and acetone. Then, theTiO_2_ compact layer was deposited on the substrates by spin coating at 3000 rpm for 1 min using a TiO_2_ precursor that was prepared by mixing 0.2 M titanium(IV) isopropoxide (TTIP) and 0.1 M hydrochloric acid (HCl) 37% in anhydrous ethanol and then heated at 500 °C. After cooling it down to room temperature, the mesoporous TiO_2_ layer was deposited by spin coating at 5000 rpm for 30 s using a TiO_2_ paste obtained with the Pechini method.^37^ The solutions were prepared from a titanium(IV) isopropoxide:citric acid:ethylene glycol solution with a molar ratio of 1:4:16, respectively. The TiO_2_ paste was prepared by heating the ethylene glycol to 70 °C and then adding the titanium(IV) isopropoxide into ethylene glycol. Finally, citric acid monohydrate was added, and the temperature was increased to 90 °C. The solution was stirred at this temperature until its color becomes clear and diluted in ethanol. The mesoporous TiO_2_ layer, which was obtained with the Pechini method, was dried at 100 °C and was gradually heated to 500 °C.

#### 2.2.2 Substitution of Bi and Co

A total of 1 M PbI_2_/*N*,*N*-dimethylformamide solution was first spin-coated onto the porous TiO_2_ at 3000 rpm for 30 s and the solution was kept at 70 °C. Then, a 50 mg/mL of MAI/ IPA solution was immediately spin-coated on the PbI_2_ film at 3000 rpm for 30 s and heated to around 100 °C for 60 min to form the perovskite layer. Two different solutions were formed by dissolving CoI_2_ and BiI_3_ precursors separately in DMF and mixed with PbI_2_/DMF solution, and these solutions were heated at 70 °C for 30 min. Prepared Co^2+^- and Bi^3+^-substituted PbI_2_ solutions were rotated and coated onto the TiO_2_-coated substrates at 1600 rpm for 30 s and then heated at 70 °C for 15 min. CH_3_NH_3_I was dissolved in isopropanol, spin-coated onto the Co^2+^- and Bi^3+^-substituted PbI_2_-based substrates, and heated to around 100 °C for 60 min to obtain CH_3_NH_3_Pb_0,9_Co_0,1_I_3_ and CH_3_NH_3_Pb_0,9_Bi_0,1_I_3_ films.

## 3 Methods

The surface morphologies of the samples were studied by Nova NanoSEM 650-FEI field emission scanning electron microscopy (FE-SEM). The thickness of mesoporous TiO_2_ layers was measured by a cross-sectional SEM analysis. The optical band gap of electrodes was determined via Tauc plot by using UV–vis spectra (Jasco V-730 UV–visible/NIR spectrophotometer). X-ray diffraction (XRD) patterns were obtained by using a PANalytical EMPYREAN with Cu Kα radiation at a scan rate of 0.1 s/step and a step size of 0.3 degrees. The electrochemical performance of supercapacitors was investigated by cyclic voltammetry (CV), electrochemical impedance spectroscopy (EIS), and galvanostatic cycling with potential limitations (GCPL) techniques in a two-electrode setup using a BioLogic VMP 300 multipotentiostat at room temperature. CV curves at various scan rates from 10 to 200 mV/s were recorded over the voltage range from 0 to +0.6 V. A sinusoidal signal of 10 mV was applied in the frequency range from 10 mHz to 1 MHz for the EIS measurements.

## 4 Electrolyte Preparation and Perovskite-Based SC Design

Quasi-solid-state electrolytes were prepared by preparing a homogeneous solution of 1 g PVA in 10 mL of chlorobenzene solution at 80 °C. After cooling down the solution to room temperature, 0.8 g of KOH was added into the solution, and then the temperature was again increased to 80 °C to ensure complete dissolution. PVA is generally soluble in water-based solvents, but the perovskite layer is a moisture-sensitive material. Therefore, an anhydrous solvent, chlorobenzene, was preferred. The obtained electrolyte was dripped onto the perovskite layer. After the production of perovskite based thin-film electrodes, electrochemical SCs were fabricated.

**Figure 1:**
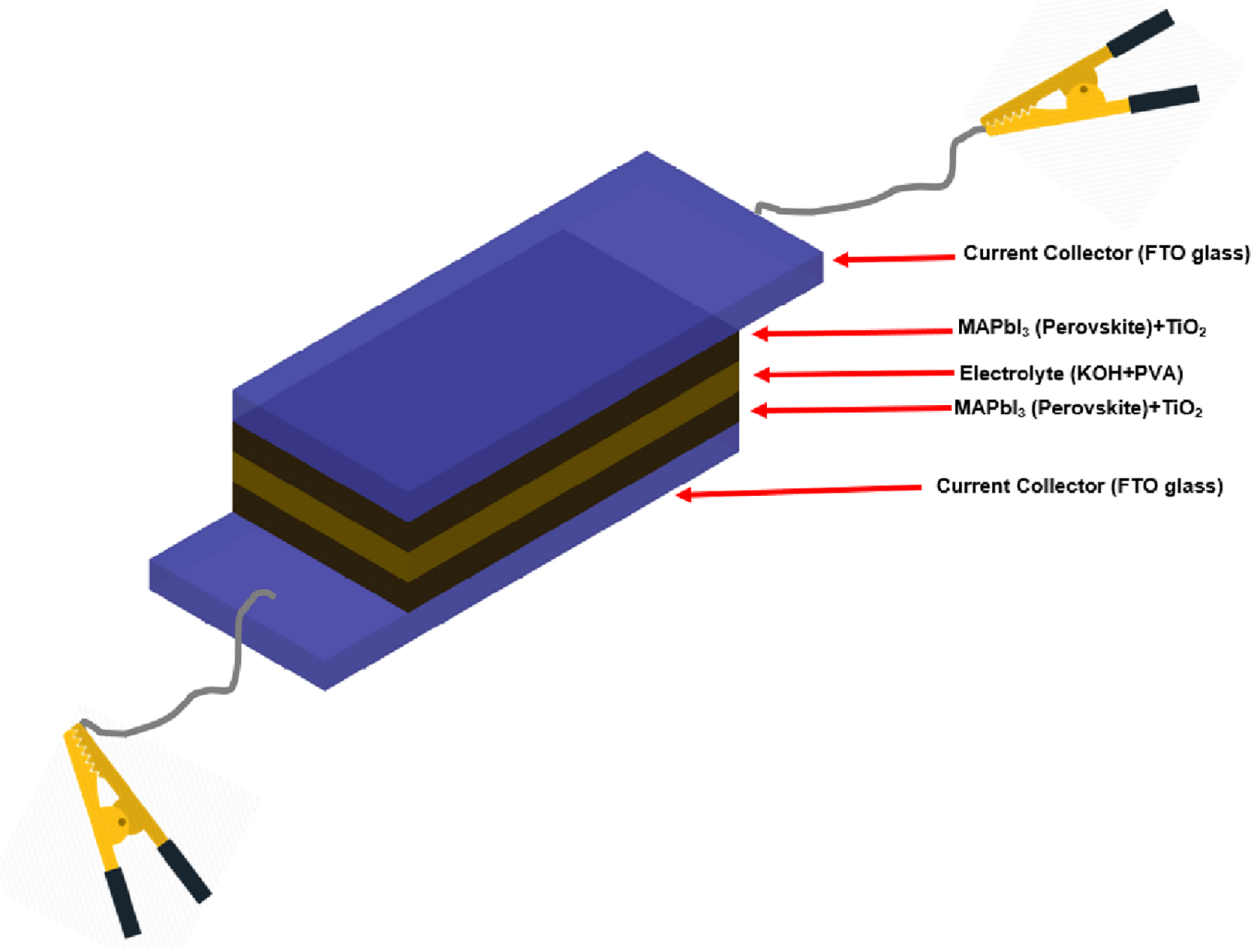
Schematic representation of the assembly of perovskite-based symmetrical SCs.

The SCs were assembled symmetrically in a glove box by sandwiching the synthesized thin-film electrodes in a quasi-solid-state electrolyte between two TiO_2_-coated FTO glasses as shown in Figure 1. The areal PVA-KOH electrolyte loading was 0.0570 g/cm^2^ for all positive and negative electrodes. The active electrode area was 1.6 cm^2^ for all SCs, and the total areal electrode loading was 0.1005 g/cm^2^.

## 5 Structural Characterization

The crystal structures and morphologies of the synthesized materials were investigated by using XRD and SEM. These analyses are complementary to the electrochemical analyses because understanding the differences in microstructures and the phase changes plays a vital role in the assessment of the mechanisms behind the different electrochemical behaviors.

**Figure 2:**
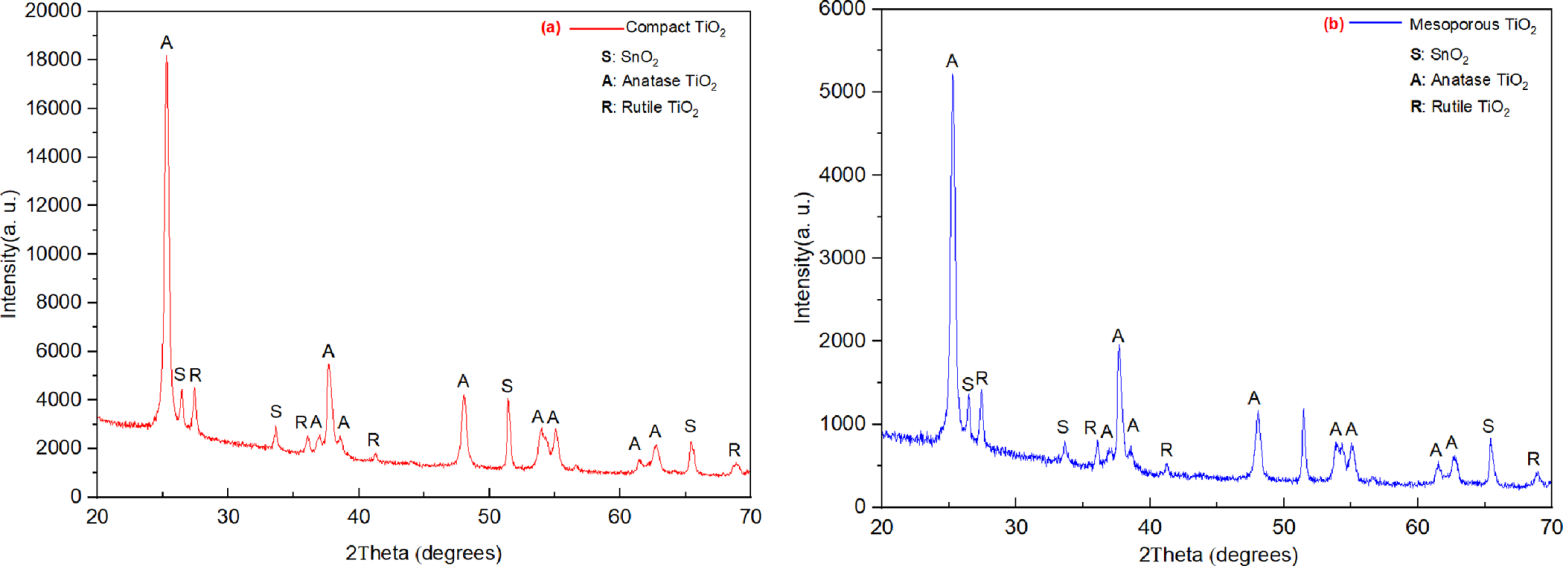
X-ray diffraction patterns of the synthesized TiO_2_ layers after annealing at 500 °C: (a) compact TiO_2_ and (b) mesoporous TiO_2_ film.

TiO_2_ rutile and anatase phases were made by spin coating and the sol–gel method. Both samples were annealed for 30 min at 500 °C. Since a more compact layer is obtained for the sample in Figure 2a, the intensity of the major anatase peak at 2θ = 25.6° is considerably higher than that of the mesoporous TiO_2_ shown in Figure 2b. In perovskite solar cells, a high surface area is critical because electrons are removed at the interface between TiO_2_ and perovskite.^38^ However, mesoporous TiO_2_ has low electrical conductivity as the structure contains a low anatase phase, so they are used with a compact TiO_2_ layer containing a more anatase phase.^39^

**Figure 3:**
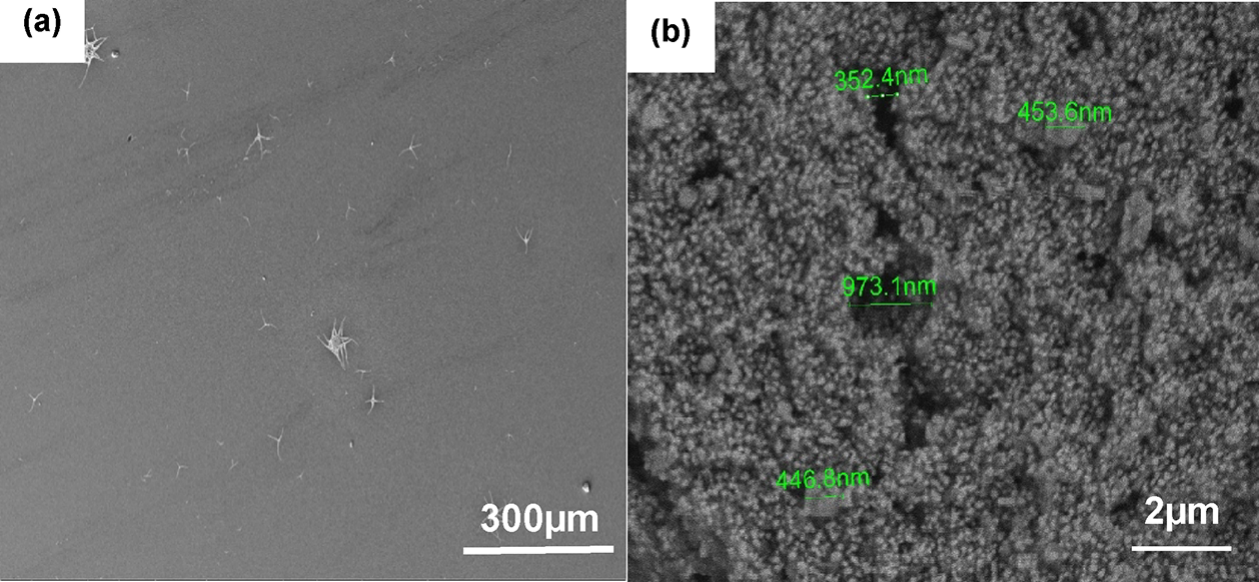
SEM images of the synthesized TiO_2_ layers after annealing at 500 °C: (a) compact TiO_2_ layer obtained by the dilution and filtering technique and (b) mesoporous TiO_2_ layer obtained by the spin coating method.

Figure 3 shows the SEM images of the synthesized TiO_2_ layers. As seen in Figure 3a, the spin-coated layer has a compact microstructure without the presence of pores and has some impurities (organic residues). However, the second layer prepared by the dilution and filtering technique using P25 powder has a mesoporous structure with the average pore size of around 663 nm and enlargement of TiO_2_ film pores are observed nearly from 353 to 973 nm (Figure 3b). Increased porosity of the mesoporous TiO_2_ layer provides higher surface area for the electrodes and provides pathways for the effective mass and charge transport.^40^

**Figure 4:**
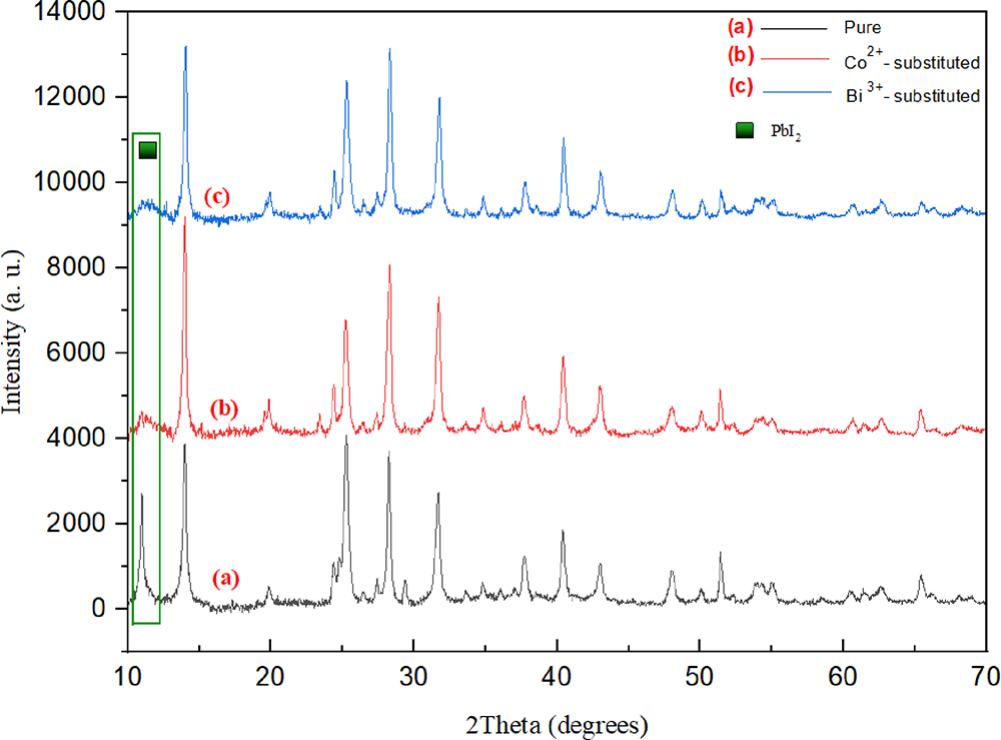
X-ray diffraction patterns of (a) pure, (b) Co^2+^-substituted, and (c) Bi^3+^-substituted perovskite films coated onto the TiO_2_ layers.

XRD patterns of of pure, Co^2+^-substituted, and Bi^3+^-substituted CH_3_NH_3_PbI_3_ perovskite films are shown in Figure 4. The strong diffraction peaks of perovskite films coated on the mesoporous and compact TiO_2_ layers were observed at 2θ = 14.1, 28.4, 31.7, and 42.1°, which correspond to the (110), (220), (310), and (330) crystal planes, respectively, and are in agreement with the literature.^41^ In addition, the intensity of the PbI_2_ peak at 2θ = 12.2° was significantly decreased in Co^2+^- and Bi^3+^-substituted samples, as depicted in Figure 4, indicating the conversion of the PbI_2_ phase into the perovskite structure. Substitution of Co^2+^ and Bi^3+^ ions led to the formation of a larger grain size of perovskite; however, it causes a decrease in the PbI_2_ grain size; hence, the perovskite phase becomes dominant.

**Figure 5:**
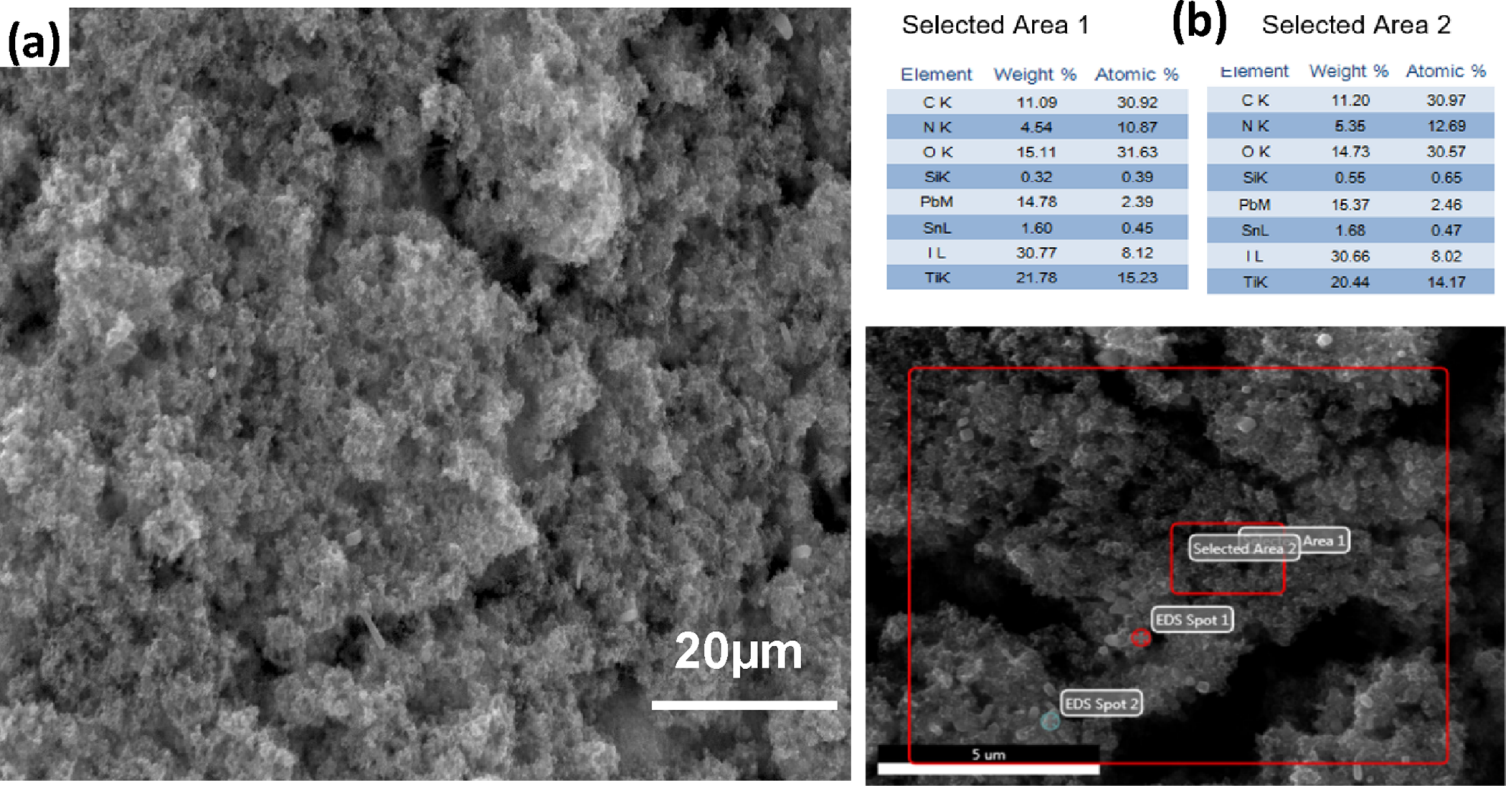
SEM cross-section image of the (a) pure CH_3_NH_3_PbI_3_ structure and (b) EDX analysis.

SEM images of CH_3_NH_3_PbI_3_ thin films and EDX analysis are shown in Figure 5a,b. The deposited perovskite thin films are homogeneous and densely packed due to the use of the double-step spin-coating process to prepare perovskite thin films instead of using the one-step spin-coating method, which is not sufficient for a complete covering of the TiO_2_ layer by the perovskite.

**Figure 6:**
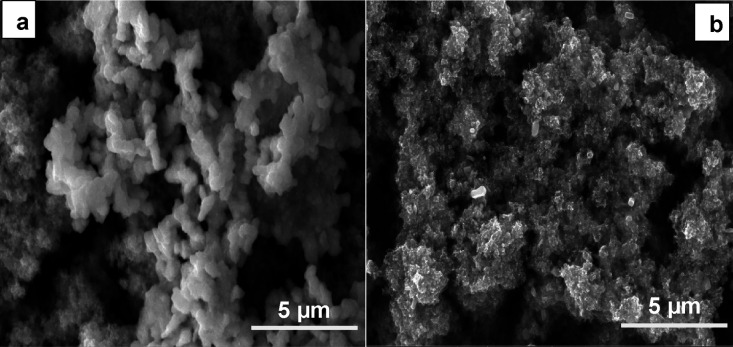
(a) SEM view of the Bi^3+^-substituted CH_3_NH_3_PbI_3_ film. (b) SEM view of the Co^2+^-substituted CH_3_NH_3_PbI_3_ film.

The surface images of the CH_3_NH_3_Pb_0,9_Bi_0,1_I_3_ and CH_3_NH_3_Pb_0,9_Co_0,1_I_3_ coated films are shown in Figure 6,b, respectively. Fewer defects are presented in the CH_3_NH_3_Pb_0.9_Co_0.1_I_3_ thin film (Figure 6b). These defects act as the leakage current pathways because CoI_2_ may act as the nuclei. Bi-containing CH_3_NH_3_PbI_3_ perovskite films (Figure 6) show a compact and smooth surface, with larger crystal grains than the undoped ones (Figure 5).

## 6 Optical Properties

The electrical conductivity and optical properties of perovskite are adjustable and controllable via band gap engineering and therefore play an important role in highly efficient SCs.^42,43^ Since the decrease in the band gap will increase the conductivity, substitution of cobalt ions at the determined level can increase the power density of the SCs. The most common technique used for measuring the optical band gap is the Tauc equation derived from UV–vis measurements, which is described in eq 1:
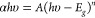
1where α is the absorption coefficient, *A* is a proportionality constant, *h* is the Planck’s constant, υ is the frequency of the incident photon, *n* is a constant that depends on the type of the transition, and *E*_g_ is the optical band gap energy of the material.

**Figure 7:**
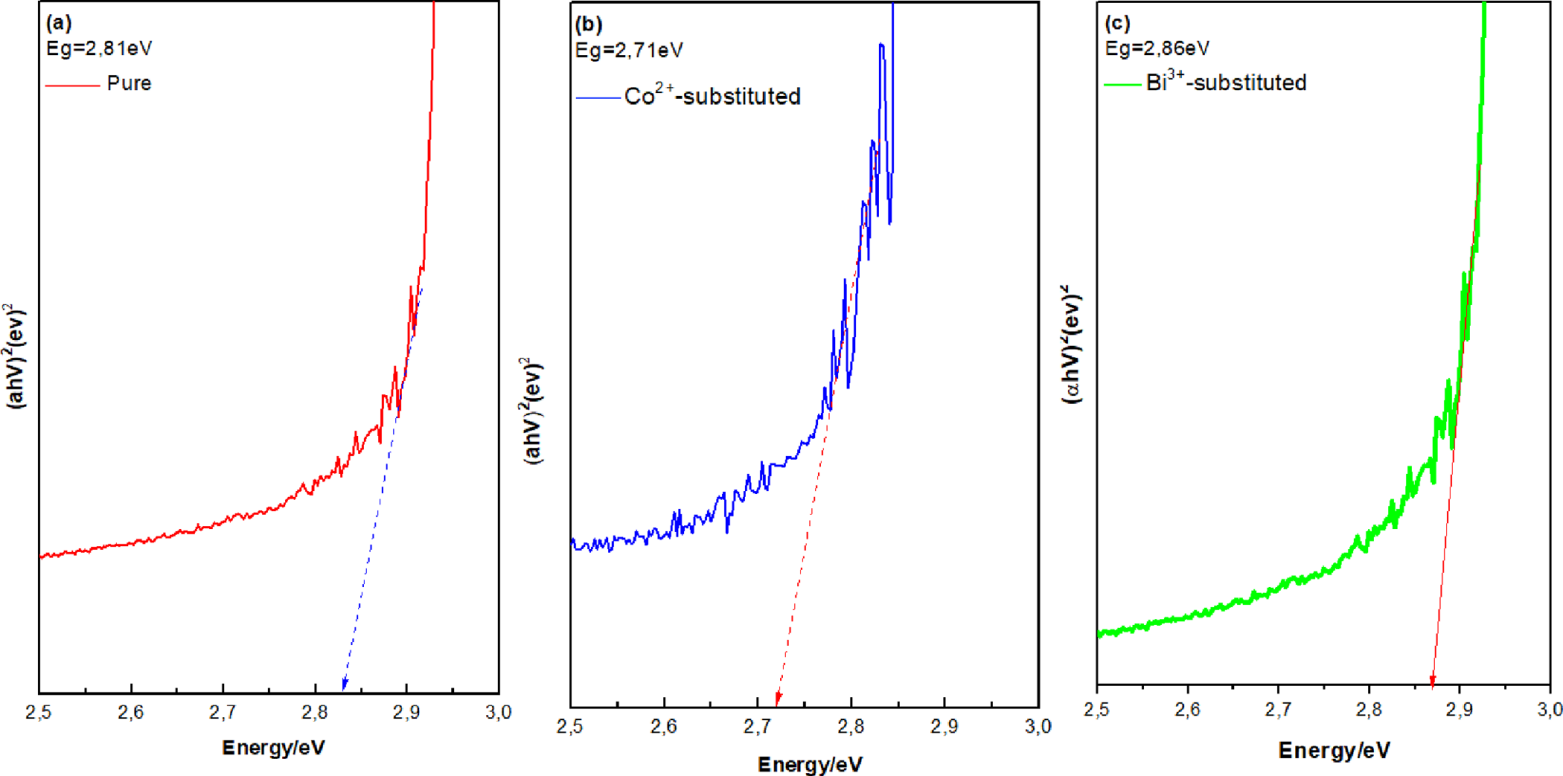
Optical band gaps calculated from UV–vis spectroscopy absorption data for (a) pure CH_3_NH_3_PbI_3,_ (b) Co^2+^-substituted CH_3_NH_3_PbI_3_ film, and (c) Bi^3+^-substituted CH_3_NH_3_PbI_3_ films.

By using the Tauc equation in the UV–vis spectra presented in Figure 7, the *E*_g_ of 2.81 eV was found for the CH_3_NH_3_PbI_3_, whereas *E*_g_ values of 2.71 and 2.86 eV were obtained for the Bi^3+^- (CH_3_NH_3_Pb_0,9_Bi_0,1_I_3_) and Co^2+^-substituted (CH_3_NH_3_Pb_0,9_Co_0,1_I_3_) perovskites, respectively. CH_3_NH_3_PbX_3_ series are adjustable in the 1.50–3.2 eV range by changing halogen ratios and organic cation types.^44^ In previous studies, the *E*_g_ of 1.56 eV was reported for the standard MAPbI_3_ perovskite films (PbI_2_ film in pure MAI solution),.^45^ For (CH_3_NH_3_)_3_Bi_2_I_9_, a wide band gap of 2.9 eV was reported, which could limit the diffusion length of carriers and mobility.^46^ The *E*_g_ values of MAPb(*Co*)I_3_ were determined to be 1.54 eV.^20^ In our study, we have obtained wide band gaps that can be attributed to two reasons: (i) Moss–Burstein (MB) effect, which occurs when the carrier concentration exceeds the edge density in the conduction band, and (ii) impurity distribution in samples.^47,48^ According to the MB effect, the semiconductor’s band gap increases when all states closer to the conduction band get to move the absorption edge to higher energy states.^49^ Also, unintentional impurities such as methylammonium iodide concentration and insoluble PbI_2_ particles in the active layer of perovskite can influence the band gap and the performance of SCs. The band gap for the ionization energy in bismuth-based perovskite tends to be higher than CH_3_NH_3_PbI_3_ because of the higher atomic number of Bi^3+^.^50^

## 7 Electrochemical Analysis

**Figure 8:**
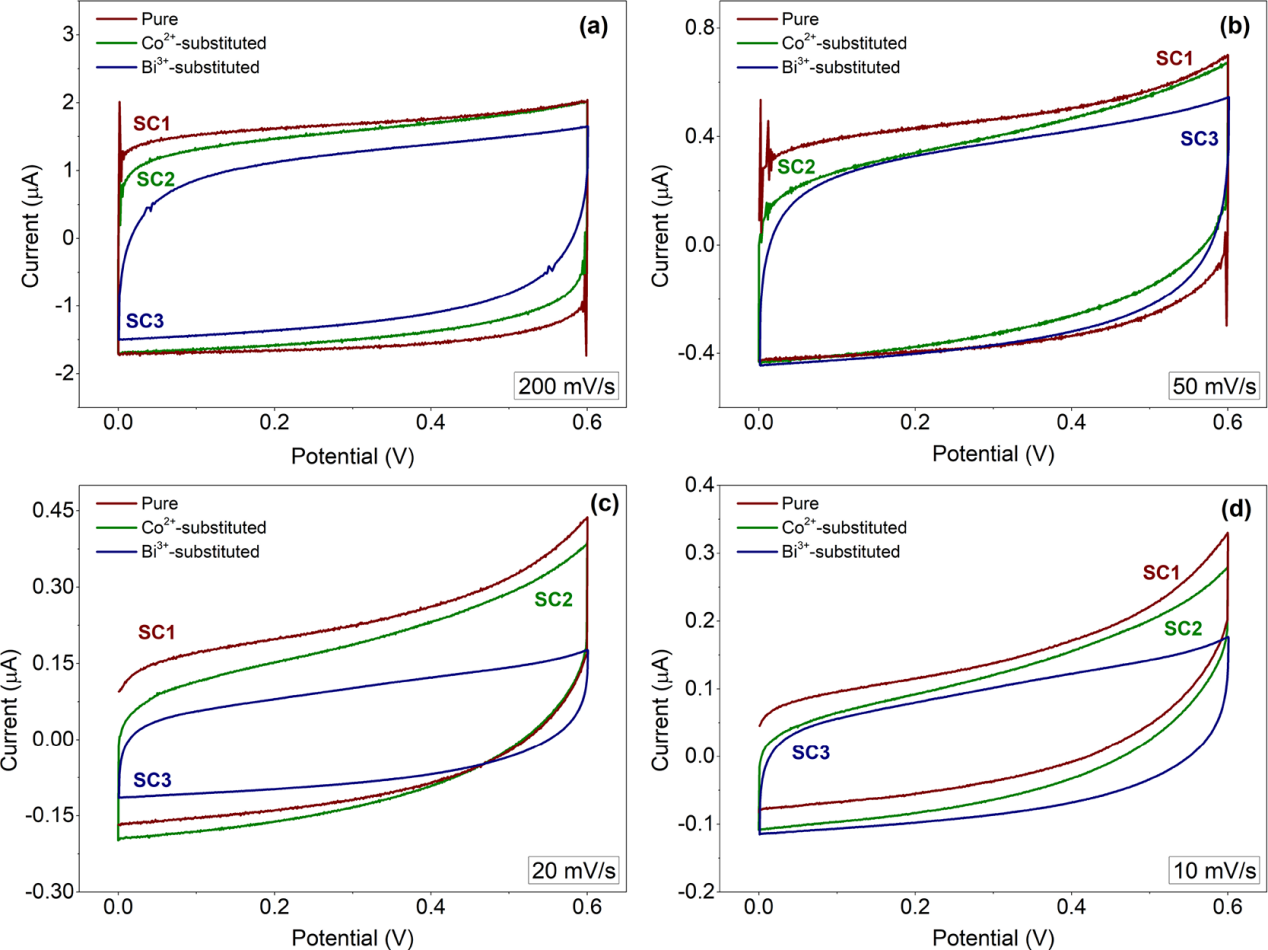
CV profiles of the pure, Co^2+^-substituted, and Bi^3+^-substituted perovskite supercapacitors in a quasi-solid-state KOH + PVA electrolyte at high and low scan rates: (a) 200, (b) 50, (c) 20, and (d) 10 mV·s^–1^.

The electrochemical properties of the fabricated all-in-one perovskite-based symmetric SCs in a quasi-solid-state PVA-KOH electrolyte were characterized by CV, EIS, and GCPL techniques. The main purpose of the use of the electrochemical tools is to understand the charge transfer processes that take place at the electrode–electrolyte interface and to investigate the effects of the substitution ions on the electrochemical behavior of the perovskite-based electrodes. Figure 8 displays the CV curves of symmetric SCs at various scan rates from 10 to 200 mV/s in the voltage range of 0 to +0.6 V. The CV curves retained their rectangular shape, which are typical EDLC curves, even at the highest scan rate of 200 mV/s, indicating a good cyclability, efficient ion transport, and good surface conductivity. From the area under the CV curves, there is no significant difference between the charge storage capacities where pure CH_3_NH_3_PbI_3_ exhibits slightly higher capacity than that of the Co^2+^- and Bi^3+^-substituted electrodes. More specifically, after calculating the area under the CV curves of SC1, SC2, and SC3 measured at 10 mV/s according to eq 2, the specific capacitances of 0.0667, 0.0672, and 0.0671 F/g were obtained for SC1, SC2, and SC3.
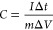
2where *C* is the specific capacitance, *I* is the discharge current, Δ*t* is the discharge time, *m* is the mass of the active area, and Δ*V* is the discharge voltage.

**Figure 9:**
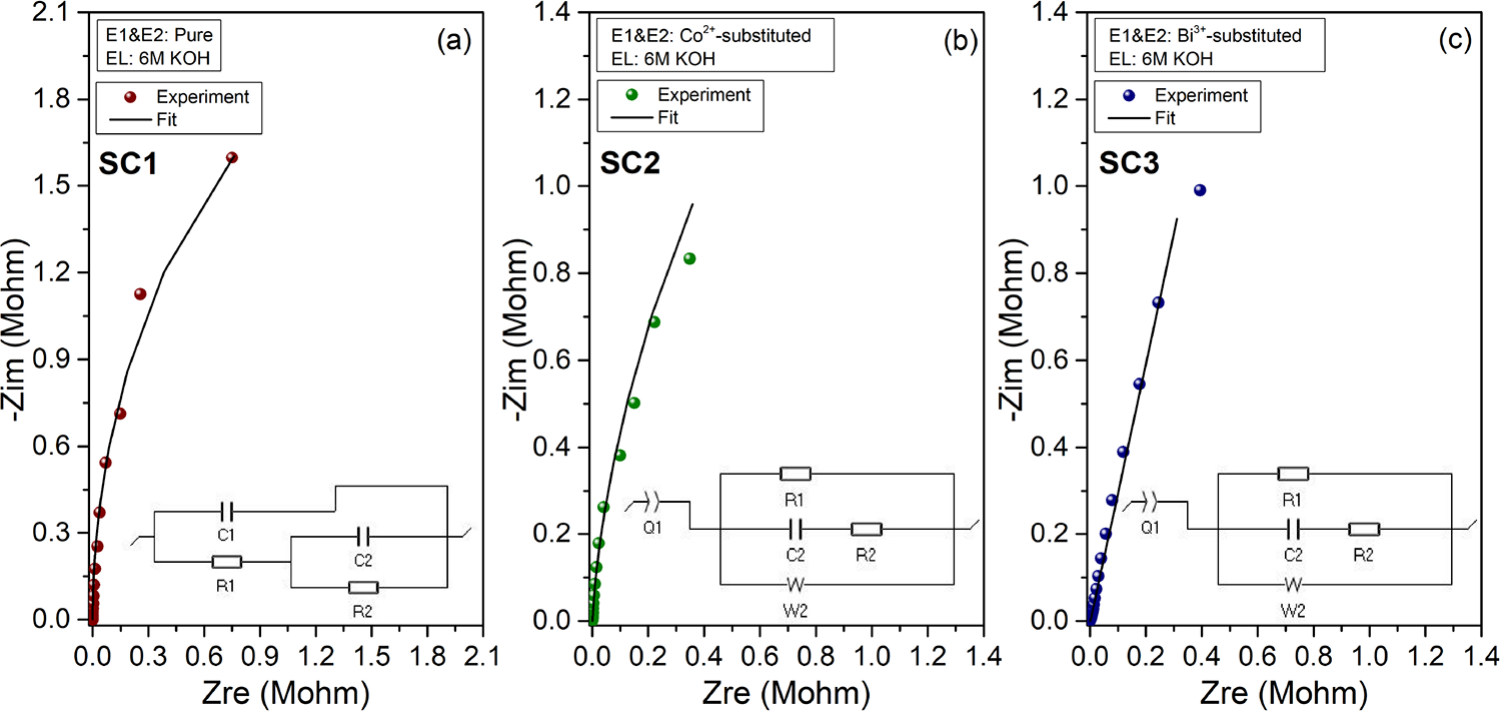
Experimental and fitted Nyquist profiles of the (a) pure, (b) Co^2+^-substituted, and (c) Bi^3+^-substituted CH_3_NH_3_PbI_3_ perovskite-based supercapacitors in a quasi-solid-state KOH + PVA electrolyte. The insets in (a)–(c) show the equivalent circuits used to fit the experimental data.

**Figure 10:**
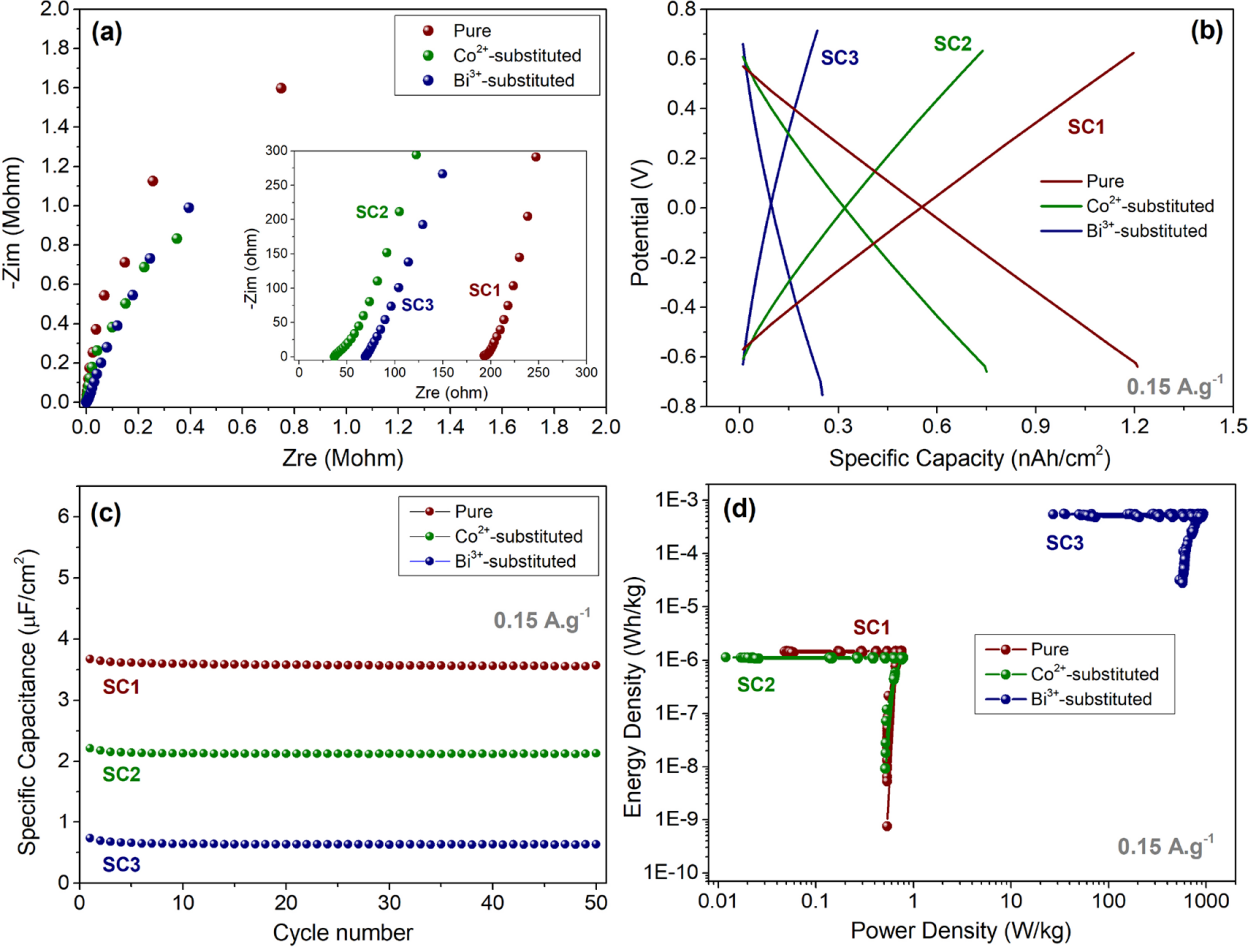
Electrochemical performance of the CH_3_NH_3_PbI_3_ perovskite-based supercapacitors: (a) comparison of the Nyquist profiles of pure, Co^2+^-, and Bi^3+^-substituted perovskite-based supercapacitors, (b) galvanostatic charge–discharge curves at a current density of 0.15 A/g, (c) cycling performance of the electrodes upon charging/discharging at a current density 0.15 A/g, and (d) Ragone plot of the fabricated supercapacitors.

Figures 9 and 10a shows the Nyquist plots and the corresponding equivalent circuits used to fit the Nyquist spectra of the pure, Co^2+^-substituted, and Bi^3+^-substituted perovskite supercapacitors in a quasi-solid-state KOH + PVA electrolyte in the frequency range of 10 mHz–1 MHz. The impedance spectra of all SCs exhibit similar behavior. There is no obvious semicircle observed at high and mid-low frequencies, and steep lines at low frequency regions represent the fast ion transfer with charge transfer resistances (*R*_CT_) of 202.8, 38.55, and 76.57 Ω for the pure, Co^2+^-substituted, and Bi^3+^-substituted perovskite SCs, respectively, revealing the effects of Co^2+^ and Bi^3+^ substitution into the CH_3_NH_3_PbI_3_ lattice on lowering the *R*_CT_.

The galvanostatic charge–discharge curves of the pure and Co^2+^-substituted perovskite-based supercapacitors are shown in Figure 10b,c at a current density of 0.15 A g^–1^ for the voltage window of 0.6 V. The observed charge and discharge curves are characteristic for EDLC type SCs. After 50 cycles, pure CH_3_NH_3_PbI_3_ delivered higher areal capacitance of 3.57 μF/cm^2^ than that of the Co^2+^- (2.13 μF/cm^2^) and Bi^3+^- (0.64 μF/cm^2^) substituted perovskite SCs, in accordance with the CV results. Figure 10d represents the Ragone plots of the fabricated SC devices. The device made of Bi^3+^-substituted perovskite electrodes exhibits an excellent power density of 934.6 W/kg at a current density of 0.15 A/g, but the energy densities are needed to be improved to provide more effective charge storage. The excellent power density of Bi^3+^-substituted perovskite electrodes can be attributed to the role of Bi^3+^ ions on hindering the rapid migration of ions at the electrode/electrolyte interface.

The reason why we performed charge–discharge measurements from −0.8 to 0.8 V is explained as follows. First, the potential window from 0 to 0.8 V is preferably used for batteries, as processes taking place are polarity dependent (negative and positive) given the separate diffusion-controlled reactions taking place at the anode and the cathode. However, the supercapacitors are independent of any polarity as they are symmetric devices. They can be charged or discharged from either of the sides under either sign of potential. We focusing on supercapacitors is the reason why we have used a voltage window of −0.8 to 0.8 V, which allows us to confirm the symmetrical operation of the capacitor unlike a battery. Second, the chemical stability of the electrolyte against anodic and cathodic reactions is one of the most important factors controlling the performance of EDLCs because it determines the maximum operational voltage. The electrolyte’s stability is typically evaluated by measuring its electrochemical potential window. This is defined as the potential difference across the electrolyte when redox reactions between the electrolyte and electrode surfaces start to occur. So, it will be convenient to apply −0.8 to 0.8 V which generates a potential difference of 1.6 V. Third, in the case of asymmetric supercapacitors, it is much easier to monitor the pseudocapacitive behavior by this kind of potential window. It seems it is contradicting with the symmetric capacitors; nevertheless, the deviations from the mirror images of the CV plots can be easily monitored even for asymmetric ones. Deviations from rectangular EDLC capacitive shapes to pseudocapacitive shapes can be monitored. The disadvantage of such scanning might be the additional energy loss. Therefore, in our next set of tests, we will carefully perform our CV tests on both (−)-to-(+) V scans and 0-to-(+) V scans and compare the results and performance. Finally, in general, one may calculate the capacitance of EDLC by *C* = *i*/*sm*, where *i* is the average current, *s* is the scan rate, and *m* is the mass of the electrode. Hence, the average current “*i*”, i.e., (*i*1 + *i*2)/2, and the electrodes in EDLC are symmetric in nature, and −0.8 to +0.8 V indicates the total operating voltage of 1.6 V. Therefore, one may evaluate the electrochemical performance of fabricated capacitors over −0.8 to +0.8 V. In the case of pseudocapacitance the type of electrolyte used is also one of the main factors for the determination of the potential window. It can fluctuate with the use of different electrolytes, such as acidic, organic, or aqueous electrolytes. The potential window is normally determined by performing CV until there is the occurrence of a hydrogen evolution reaction (HER) on the negative side and oxygen evolution reaction (OER) on the positive side, where HER and OER denote the breakdown of the electrolyte and can be shown by a sharp increase in current density. In addition, simply, the specific capacitance is decreased with increasing the scan rate, as at lower scan rates, the electrolyte ions have sufficient time to penetrate the pores of the material, while at higher scan rates, only it accumulates on the outer surface of the materials, and that is why in general, the specific capacitance drops at higher scan rates.

**Figure 11:**
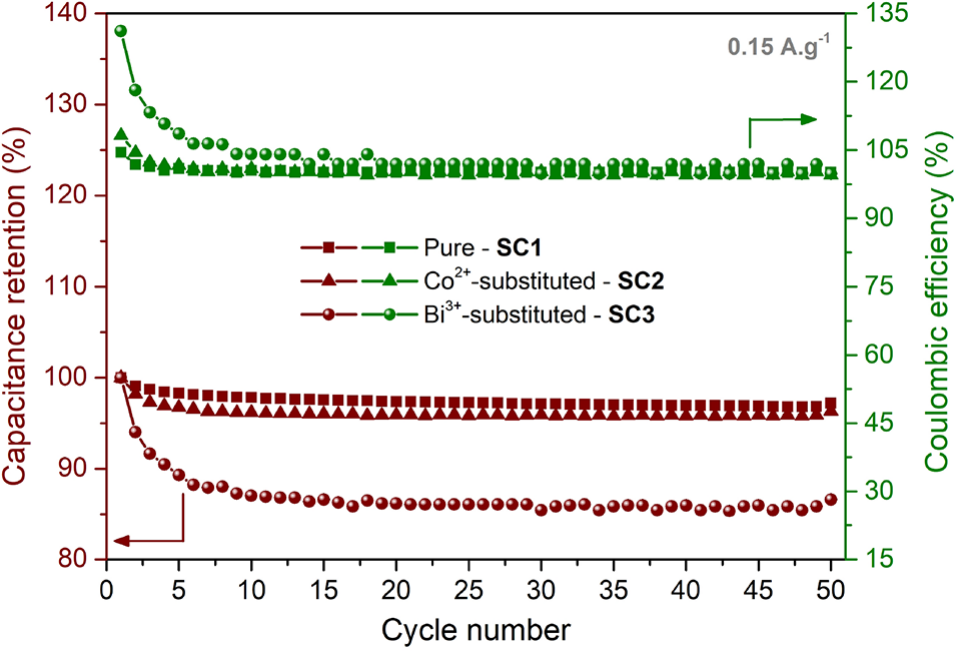
Capacitance retentions and coulombic efficiencies of the pure and Co^2+^- and Bi^3+^-substituted perovskite-based supercapacitors under repeated cycling at a current density of 0.15 A/g for 50 cycles.

As it is shown in Figure 11, the devices made of the pure, Co^2+^-substituted, and Bi^3+^-substituted perovskite electrodes retained 97.2, 96.3, and 86.6% of their initial capacitances after 50 cycles of charging and discharging with Coulombic efficiencies of 100.1, 99.5, and 99.8%, respectively, indicating good cycling capability, which are of great importance for high-performance supercapacitor devices.

## 8 Results and Discussion

In summary, the supercapacitor devices made of pure, Co^2+^-substituted, and Bi^3+^-substituted perovskite electrodes in quasi-solid-state electrolytes were assembled as symmetric two-electrode cells. The Bi^3+^ and Co^2+^ ions were partially substituted for Pb^2+^ ions in the perovskite lattice to reduce the molar fraction of Pb^2+^ and to fabricate electrochemical supercapacitors based on Co^2+^- and Bi^3+^-substituted derivatives of CH_3_NH_3_PbI_3_ to investigate the effects of ion substitution on the electrochemical performance of the SC devices. The SC device made of pure perovskite electrode demonstrated the highest areal capacitance among others, whereas its Bi^3+^-substituted derivative exhibited an excellent power density of 934.6 W/kg, which can be ascribed to the hindering effect of the Bi^3+^ substitution on the rapid migration of large ions at the electrode and electrolyte interface. All devices showed excellent charge and discharge rates with capacitance retentions of 97.2, 96.3, and 86.6% for the devices made of the pure, Co^2+^-substituted, and Bi^3+^-substituted perovskite electrodes, respectively. Further investigations on the homogeneous distribution of the electrode active materials within the active electrode area and the asymmetric design of the devices could enhance the energy density and the specific capacity of the perovskite-based electrodes for high-performance supercapacitor applications.

The rapid development of electronic devices for electrochromic supercapacitors, microelectronics, and self-healing supercapacitors will increase the need for miniature energy devices. Perovskite can be used in both photovoltaic applications and supercapacitor technology such as miniature energy devices.^51^ With this feature, the two systems can be integrated into each other. While integrated supercapacitors face great challenges, they also present many opportunities and photosupercapacitors could hold the majority of the energy storage device market.

## Supporting Information Available

Details of crystallization process and perovskite film deposition technique and additional electrochemical performance tests (cyclic voltammetry measurements, SEM images, and EDX mapping) (PDF)

## Notes

The data that support the findings of this study are available from the corresponding author.
