# Supplementary material for: All-in-One Electric Double Layer Supercapacitors Based on CH3NH3PbI3 Perovskite Electrodes
Source: ACS Omega. 2022 Dec 9;7(50):47306–16. doi: 10.1021/acsomega.2c06664 (PMC9774324; doi:10.1021/acsomega.2c06664)
Supplement: Supplementary file 1 — ao2c06664_si_001.pdf [file ao2c06664_si_001.pdf]

Supporting Information

All-in-One Electric Double Layer Supercapacitors Based on $CH_3NH_3PbI_3$ Perovskite Electrodes

Seher Güz[&], Merve Buldu-Akturk[§], Hasan Göçmez[&], Emre Erdem[§,#,ξ]

[&]Dumlupınar University, Faculty of Engineering, Department of Metallurgy and Materials Engineering, Kütahya, Turkey

[§]Sabanci University, Faculty of Engineering and Natural Sciences, İstanbul, Turkey

[#]Sabanci University, Integrated Manufacturing Technologies Research and Application Center & Composite Technologies Center of Excellence, Teknopark Istanbul, Pendik, 34906, Istanbul, Turkey.

[ξ] Sabanci University, Center of Excellence for Functional Surfaces and Interfaces for Nano-Diagnostics (EFSUN), Orhanli, 34956 Tuzla, Istanbul, Turkey.

* Corresponding Author: E-mail: emre.erdem@sabanciuniv.edu

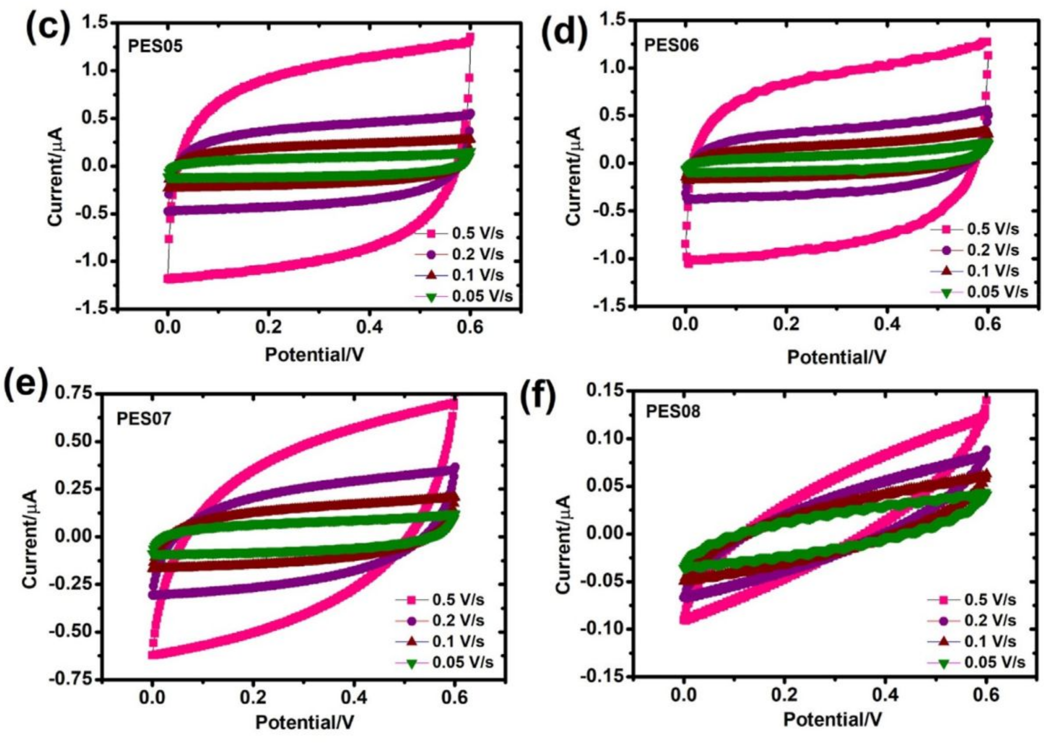

Figure S1. c) Cyclic voltammetry (CV) curves of PES05 perovskite active electrode utilizing CHLPVAKOH electrolyte without separator. d) CV curves of PES06 perovskite active electrode utilizing CHLPVAKOHMAI electrolyte without separator. e) CV curves of PES07 perovskite active electrode utilizing CHLPVAKOH electrolyte with separator. f) CV curves of PES08 perovskite active electrode utilizing CHLPVAKOHMAI electrolyte with separator.[1]

CV curves of PES05 and PES06 devices showed rectangular CV behavior across the different scan rates as shown in Figure S1 (c-d). PES07 device showed a less rectangular

CV behavior at very high scan rate of 500 mV/s (Figure 7e). PES08 device showed poor rectangular CV properties at all the chosen scan rates (Figure 7f). The PES06 device has lower current as compared to PES05. This is because of MAI poor ionic transport properties perovskite /electrolyte interface. PES05 and PES06 devices without a filter paper separator are more efficient than PES07 and PES08 devices with a separator.[1]

In this work we prevented the accumulation of MAI on the surfaces by not using a separator at the perovskite / electrolyte interface. So typical EDLC curves was obtained, even at the highest scan rate of 200 mV/s, indicating a good cyclability, efficient ion transport and good surface conductivity.

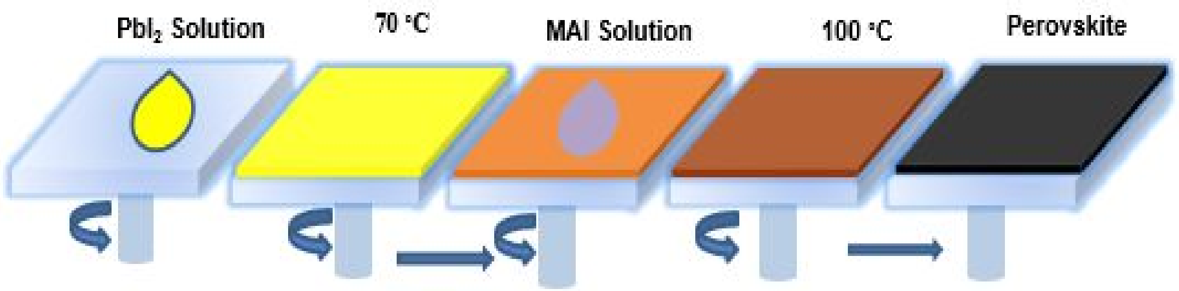

Figure S2. Production of perovskite film by two step deposition method.

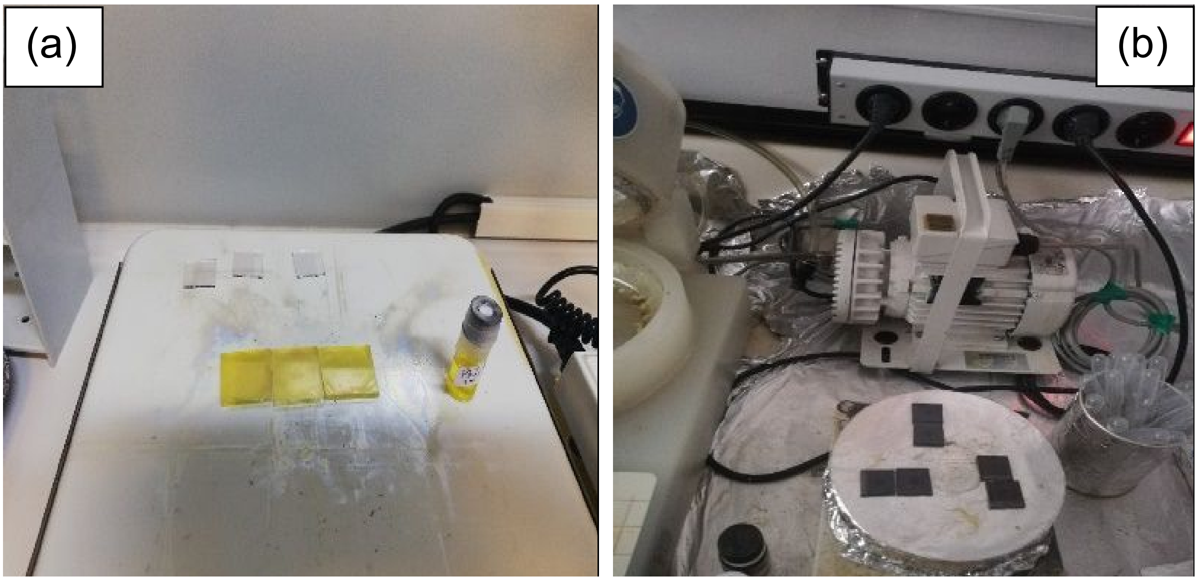

Figure S3. Crystallization process of PbI_2 (a), perovskite (b) films.

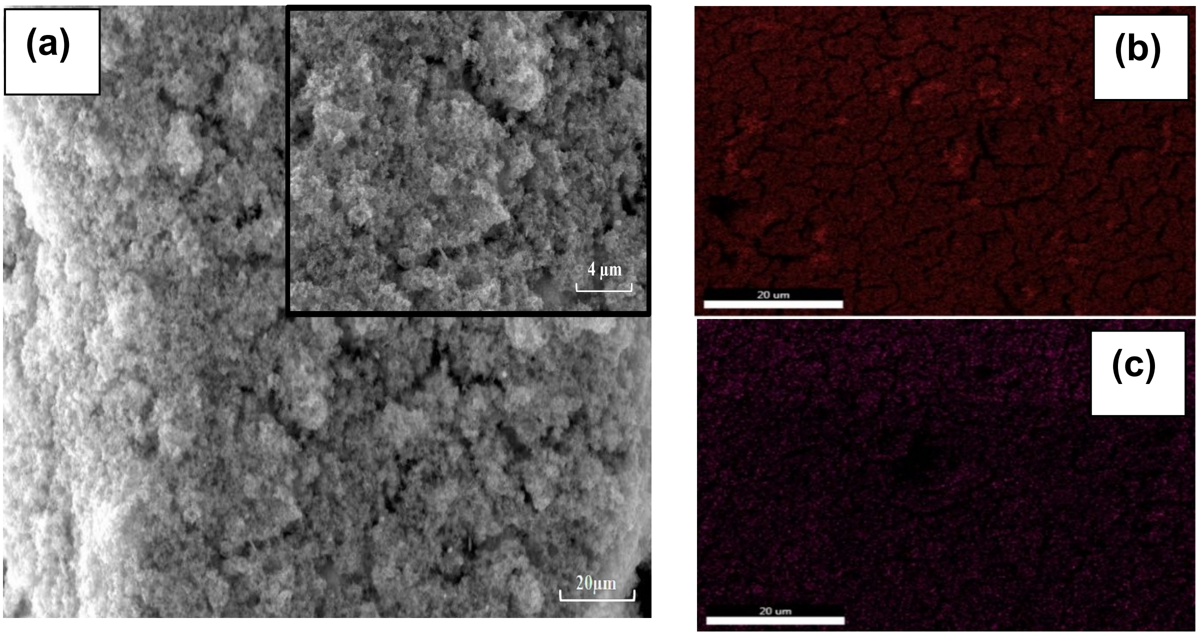

Figure S4. SEM image of pristine $CH_3NH_3PbI_3$ (a), EDX mapping of Bismuth (b), and Cobalt (c).

Reference

(1) Popoola, I.; Gondal, M.; Oloore, L.; Popoola, A.; AlGhamdi, J. Fabrication of Organometallic Halide Perovskite Electrochemical Supercapacitors Utilizing Quasi-Solid-State Electrolytes for Energy Storage Devices. *Electrochim Acta* **2020**, *332*, 135536. https://doi.org/10.1016/j.electacta.2019.135536.
